# Barriers and Attitudes toward Breast Cancer Screening among Omani Women

**DOI:** 10.31557/APJCP.2020.21.5.1339

**Published:** 2020-05

**Authors:** Mohammed Al-Azri, Kawther Al-Rubaie, Shamsa Al-Ghafri, Mustafa Al-Hinai, Sathiya Murthi Panchatcharam

**Affiliations:** 1 *Department of Family Medicine and Public Health, College of Medicine and Health Sciences, Sultan Qaboos University, Muscat, Sultanate of Oman. *; 2 *Research Section, Medical Simulation and Skills Development Centre, Oman Medical Specialty Board, Muscat, Sultanate of Oman. *

**Keywords:** Services, neoplasm, detection, screening, breast, Oman

## Abstract

**Background:**

Breast cancer (BC) is the most common cancer accounting for 24.5% of Omani female cancer. Early detection of BC through regular breast cancer screening (BCS) has been found to decrease mortality rates. The aim of this study was to identify knowledge, attitudes and barriers of Omani women toward BCS.

**Methods:**

A cross-sectional study was conducted among adult Omani women attending the Sultan Qaboos University Hospital (SQUH).

**Results:**

A total of 358 Omani women participated from 420 invited (response rate = 85.2%). Most women (92.1%) thought that BC could be cured if detected early, but less than half (46.8%) thought that they were at risk of BC if a relative had BC. The majority (81.1%) were aware that BCS was available in Oman, though less than half (48.5%) knew where to go for BCS and most (83.8%) had not undergone BCS before. The most common personal-related barriers to BCS were fear of BC diagnosis (40.8%), fear of treatment (52.1%) and embarrassment of the breast examination (46.6%). The most perceived system-related barriers to BCS were the concern over the availability of a male doctor (46.6%) or a non-Arabic speaking doctor (38.7%) and not recommended by the doctor (46.3%). Univariate binary logistic regression showed that attitudes toward BCS were influenced by their previous experiences of BCS (odds ratio [OR] = 2.28; 95% confidence interval [CI]: 1.18-4.41) and their willingness to participate in the future BCS [OR = 1.96; 95% CI: 1.14-3.37).

**Conclusion:**

Although Omani women showed an interest toward BCS, few had undergone BCS. Several cultural, practical and personal-related barriers were noted to interfere with BCS in Oman. Addressing these concerns through involving healthcare providers to reassure the women and implementing a national strategy of BCS could improve BCS screenings and early diagnosis of BC.

## Introduction

Breast cancer (BC) is the most common cancer affecting women globally, with an estimated 2.1 million women being diagnosed with BC each year. In 2018, the World Health Organisation (WHO) estimated that around 627,000 women died from BC, accounting for approximately 15% of all cancer deaths among women worldwide (World Health Organization, 2019). There are several risk factors which contribute to an increased risk of BC, including an aging population, obesity, use of menopausal hormone therapy, physical inactivity, consumption of alcohol, use of oral contraceptives, nulliparity and being of an older age when they had their first child (McPherson et al., 2000; Torre et al., 2016). Although BC mortality rates have been decreasing in many developed countries, mainly attributed to early detection and improved treatments, they continue to rise in developing countries due to changes in risk factors, limited access to routine breast cancer screening (BCS) and a lack of treatment (Bhikoo et al., 2011).

In Arabic countries, BC is the most common cancer affecting women, accounting for 13-35% of all cancer diagnoses; almost half of all women diagnosed with BC are under the age of 50, with a median age of 49-52 years, as compared to 63-years-old in developed countries (El Saghir et al., 2007). While BC incidence rates are relatively low in Arabic countries, as compared to Western countries, they are rising rapidly and the majority of women are presenting at advanced stages (Elobaid et al., 2014; Donnelly et al., 2013a). Furthermore, women in Arabic countries are facing several psychosocial, cultural and belief challenges, leading to a delay in BC diagnosis and increased risk of mortality (Al-Azri et al., 2009; Al-Azri et al; 2014). Early detection of BC through regular screening activities, such BCS, breast self-examination (BSE) or clinical breast examination (CBE), have been found to decrease mortality rates by 25-30% and greater likelihood of responding to treatment (Donnelly et al., 2013a; Key et al., 2001). 

Mammography remains the gold standard for BCS as it offers an effective means to detect BC early with reasonable sensitivity in comparison to BSE or CBE (Miller, 2001). The U.S. Preventive Services Task Force has recommended biannual mammography screening for women aged 50 to 74 years, with the decision to start prior to the age of 50 to be decided individually. Women who are at a higher risk for BC, such as those with a family history of BC, may decide to start BCS between the ages of 40 and 49 (Siu and U.S. Preventive Services Task Force, 2016). Conversely, not offering BCS to women at lower risk could improve the cost-effectiveness of screening program, reduce over-diagnosis, and maintain the benefits of screening (Pashayan et al., 2018).

In developed countries, national or sub-national BCS programs have been implemented and women in specific age ranges are regularly invited to undergo mammography (Autier et al., 2011). However, in most Arabic countries there are no centrally-organized BCS invitation or follow-up systems, with BCS remaining limited to women who are either self-motivated to take part or for those who are referred by doctors (El Saghir et al., 2007). In some Arabic countries BCS is available and free of charge for women over the age of forty, though previous studies have indicated that participation rates are low (Elobaid et al., 2014; Donnelly et al., 2013). Generally, there are low awareness levels of the importance of BCS among women in Arabic countries, due to a lack of referrals by doctors, as well as fear or embarrassment due to the physical examination (Donnelly et al., 2013a).

Oman is a developing Arabic country located in the south-eastern coast of the Arabian Peninsula. BC is the most prevalent cancer in Oman, with an increased annual incidence from 53 in 1996 to 178 in 2015 (Ministry of Health, 2015). In Oman, BC accounts for 24.50% of all cancers affecting Omani women and 12.79% of all cancer diagnoses (Kumar et al., 2011). Similar to other Arabic countries, the majority of women in Oman are diagnosed with BC at a younger age (median age of 49 years) and present at an advanced staged at the time of diagnosis, compared to Western countries, which have a five-year survival rate of 63% (Donnelly et al., 2013a; Ministry of Health, 2015; Kumar et al., 2011).

There are several social and cultural barriers contributing to the delay of BC diagnosis in Oman, particularly among vulnerable groups, such as women who are unmarried, widowed, divorced, or separated (Al-Azri et al., 2013; Al-Azri et al., 2014). Women who are diagnosed with BC are referred for treatment (surgery, chemotherapy, radiotherapy) or palliative care at two main hospitals in the country, both in Muscat, the capital city of Oman: the National Oncology Centre at the Royal Hospital and the Oncology unit at the Sultan Qaboos University Hospital (SQUH).

The National Oncology Centre is affiliated with the Oman Cancer Association (OCA), a non-governmental organisation which offers Mobile Mammography Unit (MMU) BCS services for Omani women over the age of 40. The MMU travels around the country twice a year to reach the maximum possible number of women in the community. All potential BC patients are fast-tracked towards specialists for consultation. To date, the MMU has screened more than 19,000 Omani women since it was launched in November 2009 (Oman Cancer Association, 2019). To the best of our knowledge, there has never been a study conducted in Oman to identify the knowledge, attitudes and barriers of Omani women toward BCS.

## Materials and Methods

*Tools used to measure knowledge of BC and attitudes toward BCS*

A structured questionnaire was developed from the currently available literature on similar studies conducted in Arabic countries (Donnelly et al., 2013a; Donnelly et al., 2013b; Kawar, 2013). The questionnaire included five sections: socio-economic characteristics of the participants (such as age, occupation, educational level, income); general knowledge of BC and the awareness of BCS in Oman (BC risk factors, availability of BCS, information about BCS); perceptions and attitudes toward BCS (including painfulness, dangerousness, effectiveness); personal-related barriers toward BC screening (worry, trust, embarrassment, religious beliefs); system-related barriers to BCS (accessibility, appointments, gender of the doctor). 

The questionnaires were translated into Arabic and back-translated into English. Each section of the questionnaire was tested for reliability using Cronbach’s alpha (α) among the first 50 participants. Based on the standardized items, the Cronbach’s alpha (α) for each section was as follows: general knowledge of BC and awareness of BCS, the Cronbach’s alpha (α) was 0.91; perceptions and attitudes toward BCS, Cronbach’s alpha (α) was 0.88; system-related barriers to BCS, Cronbach’s alpha (α) was 0.70; and for personal-related barriers, Cronbach’s α was 0.81.

*Setting of the study*

SQUH is a tertiary teaching hospital and receives patients from other government hospitals located from all over Oman, local health centres (LHCs) and other private hospitals. SQUH is also a training hospital for undergraduate medical students and postgraduate clinicians of varying medical and surgical specialities and subspecialties of the Oman Medical Specialties Board. SQUH was chosen to collect the data for this study as it allowed for women from various heterogeneous groups to be included, with most of the participants coming from different regions of Oman, it also allowed for convenient data collection within a specific timeframe. 

*Recruitment of participants*

This cross-sectional study was conducted from 1st January 2018 to 31st March 2018. Two female medical students were trained on how to administer the questionnaire, in order to distribute and collect the data from the study’s participants. All adult female Omani women (age >18 years) attending SQUH during the study period were invited to participate in the study. The aim of the study was explained to the women and they were given the option to participate or to opt out. Women who agreed to participate were asked to sign a consent form and they were given the questionnaire to complete. For illiterate women, the questionnaire was introduced to them by the medical students. Women who were very ill or emergency cases were excluded. 

*Sample size calculation*

There have been no previous studies conducted in Oman to investigate the knowledge, attitudes and barriers for BCS among Omani women. Therefore, for the sample size calculation, it was assumed that Omani women had a BCS knowledge level of 50%. Thus, with a precision level of 5% and a desired confidence level of 95%, it was determined that around 355 women were required. 

*Statistical analysis*

Data were analysed using the Statistical Package for the Social Sciences (SPSS), version 22 (IBM Corp., Chicago, Illinois). For descriptive statistics, categorized variables were presented as numbers, percentages and mean, standard deviations for interval variables. The Chi-squared test (*χ*^2^) were performed to determine the associations between categorical dependents and categorical predictors. A univariate binary logistic regression analyses was carried out for the factors related to perceptions of BCS (dichotomised responses: yes/no). A P value of <0.05 was considered to be significant. 

## Results

*Socio-demographic characteristics of participants*

A total of 358 women participated in this study, from a total of 420 invited (response rate = 85.2%). Their ages ranged from 18-70 years. The overall mean age of the women was 30.1 years and median was 28.0 years. The majority of women (48.8%) were between the ages of 25 and 40. Most women (52.8%) were married and most came from Al Batina (33.6%) and Muscat (30.3%). Most of the women had completed a university and postgraduate education (40.79%) and large number (41.6%) stated that they had a family history of cancer (Table 1).

The majority of women (300; 83.8%) stated that they have not undergone BCS before, and most (284; 81.1%) stated that they would like to have BCS in the future, with many (277; 77.6%) stating that they heard of BSE and only half (182; 50.8%) stating that they had done a BSE. 

*Participants’ knowledge of BC and BCS*

**Figure 1:**
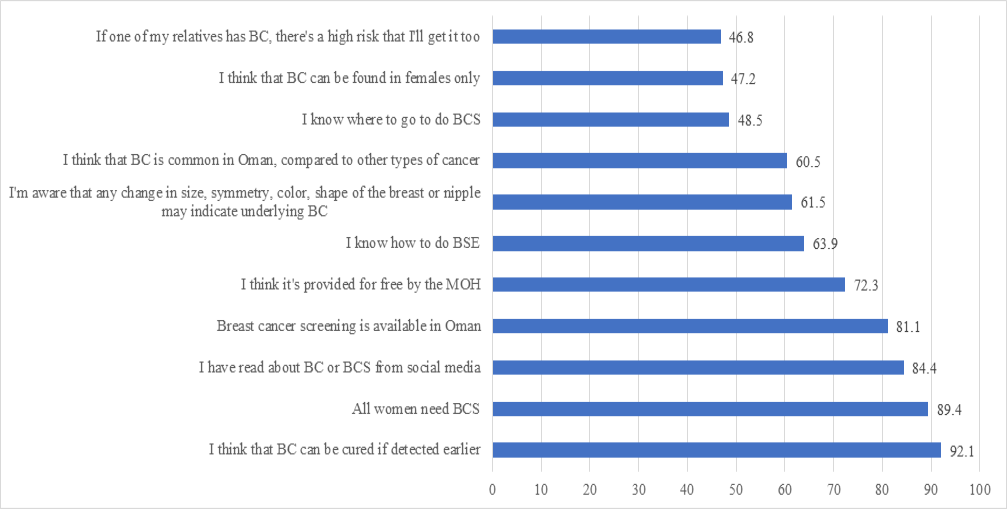
Participants’ Knowledge of BC and BCS. BC, breast cancer; BCS, breast cancer screening; BCE, breast cancer examination

More than half of the women (60.5%) knew that BC was the most common type of cancer in Oman. The majority of the women (92.1%) thought that BC could be cured if detected early and more than half (61.5%) were aware that any change in the size, symmetry, colour or shape of their breast or nipples may indicate BC. However, less than half of the women (46.8%) thought that they were at risk of BC if a relative had had BC, with more than half (63.9%) stating that they can do BSE (Figure 1).

The majority of women (81.1%) stated that they were aware that BCS was available in Oman, that they believed that all women should undergo BCS (89.4%) and that BCS is provided free of charge by the Ministry of Health (MOH) (72.3%). However, less than half of them (48.5%) stated that they knew where to go to get BCS, and the majority (84.4%) had obtained their BCS knowledge from social media (Figure 1). 

*Attitudes and perception of participants toward BC and BCS*

**Figure 2:**
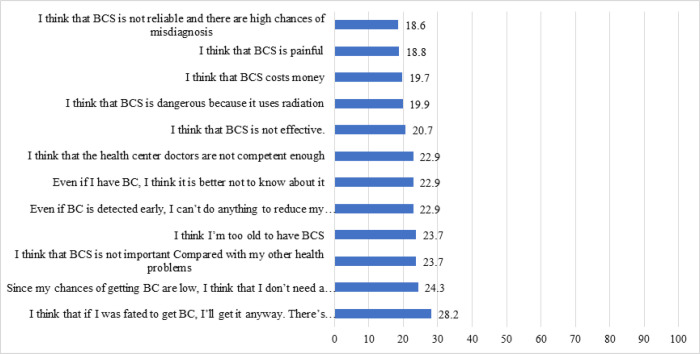
Participants’ Perceptions of BCS. BC, breast cancer; BCS, breast cancer screening; BCE, breast cancer examination

Few women agreed with the negative perceptions of BCS, including that if they were fated to be diagnosed with BC, they would get it (28.2%); that there was no need to do BCS as the chance of being diagnosed with BC was low (24.3%); that BCS was not important in compared to other health issues (23.7%); that they were too old to get BCS (23.7%); that nothing could be done to reduce the chance of death from BC even if detected early (22.9%); that it was better not to know if they had BC, even if diagnosed (22.9%); doctors in LHCs are not competent enough to diagnose BC (22.9%); BCS is not effective in diagnosing BC (20.7%); BCS is dangerous because it uses radiation (19.9%); BCS is costly (19.7%); BCS is painful (18.8%); and BCS is not effective as there is a chance of misdiagnosis (18.6%) (Figure 2).

*The perceived personal-related barriers of participants toward BCS*

**Figure 3:**
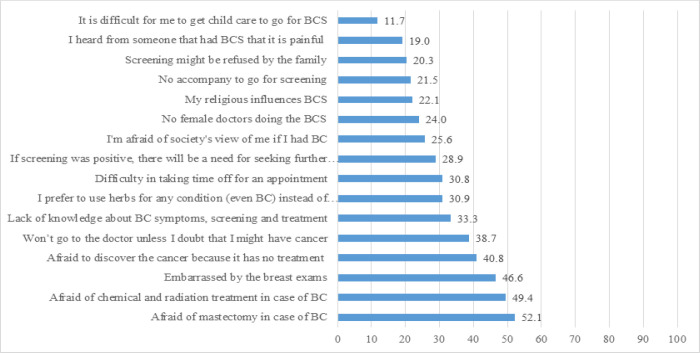
Perceived Personal-Related Barriers of Participants Toward BCS. BC, breast cancer; BCS, breast cancer screening; BCE, breast cancer examination

The most common perceived personal-related barriers of BCS was worrying about a mastectomy if BC was diagnosed (52.1%), followed by worrying about treatment (chemotherapy and radiotherapy) (49.4%); being embarrassed of the BE (46.6%); being worried about a diagnosis of BC due to no treatment (40.8%); reluctance to see a doctor unless there is a doubt of BC (38.7%); a lack of knowledge of BC symptoms, screening and treatment (33.3%); preference to use herbal medication even if BC is diagnosed (30.9%); difficulty in getting time from work for BCS appointments (30.8%); being worried about additional tests if BC is diagnosed (28.9%); negative views from society of being a BC patient (25.6%); lack of a female doctor to do the BCS (24.0%); religious beliefs which interfere with BCS (22.1%); lack of a companion when going for BCS (21.5%); being worried that their family might refuse for them to receive BCS (20.3%); the painfulness of BSC (19.0%); and difficulty in having time to attend BCS due to being busy with childcare (11.7%) (Figure 3).

*The perceived system-related barriers of participants toward BCS*

**Figure 4:**
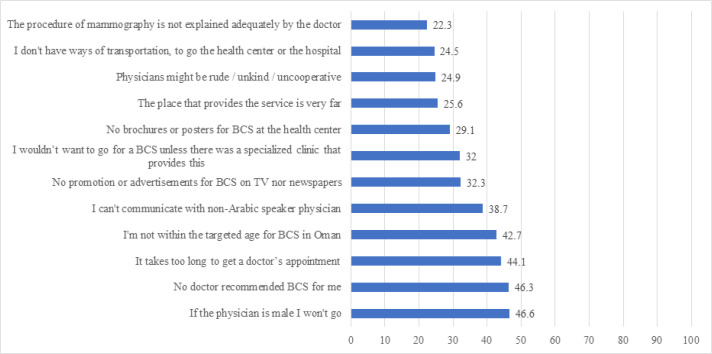
Perceived System-Related Barriers of Participants Toward BCS. BC, breast cancer; BCS, breast cancer screening; BCE, breast cancer examination

The most common systems-related barriers to BSC were that they would not attend BCS if done by male doctor (46.6%); that BCS was not recommended by their doctor (46.3%); they were worried about long appointment times (44.1%); they were not at the targeted age for BCS (42.7%); being worried about not finding non-Arabic doctor, having difficulty in communicating (38.7%); no promotion or advertising for BCS on the TV or newspaper (32.3%); lack of specialized BCS clinics (32.0%); a lack of brochures or posters about BCS in local health centres (29.1%); the BCS service is very far (25.6%); the doctors do not readily order BSC (24.9%); a lack of transportation to the site of BCS (24.5%); and the mammogram was not explained well by the doctor (22.3%) (Figure 4). 

*The association between participants’ perception of BCS and their responses*

The multinominal regression analyses indicated that women who underwent BCS in the past were significantly more likely than those who had not in to believe that: they did not need to get a BCS as their chance of having BC was low (odds ratio [OR] = 2.38; 95% confidence interval [CI]: 1.21–4.69; P = 0.012); they will definitely get diagnosed with BC if they are fated to, as nothing can change fate (OR = 2.28; 95% CI: 1.20–4.35; P = 0.012); they are too old to get BC (OR = 2.28; 95% CI: 1.18–4.41; P = 0.014); BCS is a painful procedure (OR = 2.46; 95% CI: 1.39–4.36; P = 0.002); BCS costs money (OR = 2.04; 95% CI: 1.14–3.66; P = 0.016); BCS is not reliable, with a chance of misdiagnosis (OR = 3.02; 95% CI: 1.65–5.53; P < 0.001); and the doctors in the LHCs are not competent enough to do a BCE (OR = 2.03; 95% CI: 1.15–3.58; P = 0.015) (Table 2). 

Multinominal regression analyses indicated that that women who are planning to undergo BCS were significantly more likely than women who were not to believe that they did not need BCS as their chance to get BC was low (OR = 1.93; 95% CI: 1.12–3.33; P = 0.017); that they will get BC if they are fated to, because nothing can be done to change fate (OR = 1.74; 95% CI: 1.01–2.99; P = 0.046); and that they are too old to have BC (OR = 1.96; 95% CI: 1.14–3.37; P = 0.015) (Table 2).

**Table 1:** Socio- Demographic Characteristics of the Participants

Variables	n	%
Age in years (n = 338)		
<25	122	36.1
25-40	165	48.8
>40	51	15.1
Marital status (n = 356)		
Single	156	43.8
Married	188	52.8
Divorced/Widowed	12	3.4
Region of origin (n = 357)		
Muscat	108	30.3
A’dhahira	26	7.3
Al Batina	120	33.6
A’dakhilia	57	16.0
Al Wusta	3	0.8
A’Sharqia	41	11.5
Dhofar	1	0.3
Musandam	1	0.3
Educational level (n = 357)		
Illiterate	4	1.1
No formal education	12	3.4
School education	117	32.7
Diploma	79	22.1
College & above	145	40.7
Occupation (n = 353)		
Employed	106	30.0
Unemployed	168	47.6
Student	79	22.4
Family income in OMR (n = 344)		
≤500	77	22.4
501-1000	138	40.1
1001-2000	90	26.2
>2000	39	11.3
Smoking status (n = 358)		
Yes	1	0.3
No	356	99.4
Was but stopped	1	0.3
Drinking alcohol (n = 357)		
Yes	-	-
No	351	99.4
Was but stopped	1	0.3
Family history of cancer (n = 358)		
Yes	149	41.6
No	209	58.4

**Table 2:** Association between Risk Factors and Previous Experience of BCS, as Well as Future Practices of Participants

Perceptions on breast cancer screening	Has done BCS		Unadjusted		BCS in future	Unadjusted	
			*P*-value	Odds ratio		*P*-value	Odds ratio
I think that BCS is not important in comparison with my other health needs.	Yes	46 (79.3)	0.081	1.83 (0.93 – 3.62)	203 (71.5)	0.138	1.53 (0.87 – 2.68)
	No	203 (67.7)		1	41 (62.1)		1
I don’t think that I need BCS as my chance of getting BC is low.	Yes	46 (79.3)	0.012	2.38 (1.21 – 4.69)*	191 (67.3)	0.017	1.93 (1.12 – 3.33)*
	No	185 (61.7)		1	34 (51.5)		1
I think that if I am fated to get BC I’ll get it anyway. There’s nothing I can do to change my fate.	Yes	43 (75.4)	0.012	2.28 (1.20 – 4.35)*	177 (62.8)	0.046	1.74 (1.01 – 2.99)*
	No	171 (57.4)		1	32 (49.2)		1
Even if BC is detected early, I can’t do anything to reduce my chances of dying from it.	Yes	41 (70.7)	0.228	1.46 (0.79 – 2.69)	180 (63.4)	0.969	0.99 (0.57 – 1.73)
	No	187 (62.3)		1	42 (63.6)		1
Even if I have BC, I think it is better not to know.	Yes	45 (77.6)	0.137	1.65 (0.85 – 3.21)	201 (70.8)	0.258	1.38 (0.79 – 2.43)
	No	203 (67.7)		1	42 (63.6)		1
I think that BCS is not effective.	Yes	45 (77.6)	0.072	1.84 (0.95 – 3.56)	198 (69.7)	0.098	1.59 (0.92 – 2.77)
	No	196 (65.3)		1	39 (59.1)		1
I think I’m too old to have BC.	Yes	45 (77.6)	0.014	2.28 (1.18 – 4.41)*	186 (66.2)	0.015	1.96 (1.14 – 3.37)*
	No	179 (60.3)		1	33 (50.0)		1
I think that BCS is painful.	Yes	34 (58.6)	0.002	2.46 (1.39 – 4.36)*	121 (42.8)	0.078	0.60 (0.33 – 1.06)
	No	109 (36.6)		1	20 (30.8)		1
I think that BC is dangerous because it uses radiation.	Yes	29 (50.0)	0.203	1.44 (0.82 – 2.54)	121 (42.8)	0.527	1.20 (0.69 – 2.08)
	No	122 (40.9)		1	25 (38.5)		1
I think that BCS costs money.	Yes	37 (63.8)	0.016	2.04 (1.14 – 3.66)*	146 (51.8)	0.072	1.65 (0.96 – 2.85)
	No	138 (46.3)		1	26 (39.4)		1
I think that BCS is not reliable and there is a high chance of misdiagnosis.	Yes	39 (68.4)	<0.001	3.02 (1.65 – 5.53)**	135 (48.2)	0.084	0.61 (0.35 – 1.07)
	No	124 (41.8)		1	24 (36.4)		1
I think that the health centre doctors are not competent enough to do BCE.	Yes	29 (50.0)	0.015	2.03 (1.15 – 3.58)*	102 (35.9)	0.871	1.05 (0.60 – 1.84)
	No	99 (33.0)		1	23 (34.8)		1

*P < 0.05; **P < 0.001; BC, breast cancer; BCS, breast cancer screening; BCE, breast cancer examination

## Discussion

To the best of our knowledge, this is the first study conducted in Oman to identify Omani women’s knowledge levels and altitudes toward BCS. The majority of women in our study were aware that BC can be cured if detected early, but only around half of them were aware that any change in the size, colour or shape of the breast or nipple may indicate BC. Although the Omani women included in this study, were aware of the importance of early cancer detection and the importance of BCS, yet the majority presented at advance stages compared to Western countries (III and IV) (Kumar et al., 2011). Previous studies have shown that the majority of women in Oman still lack knowledge and awareness of BC symptoms (Renganathan et al., 2014; Al-Azri et al., 2018). 

Although most women in the study were aware of the availability of BCS in Oman, had heard of BSE and thought that women should undergo BCS, only 16.2% of them stated that they had undergone BCS and around half said they had done a BSE. A previous study conducted among Arabic women in Palestine showed that more than 70% had never undergone a mammogram or CBE (Azaiza et al., 2010). Knowledge of the benefits of BCS is an important determinant behaviour to attend BCS, as lack of such knowledge can act as a barrier (Azami-Aghdash at al., 2015). Arabic women who underwent BCS or BSE were more likely to be educated, expressed less personal barriers, low fatalism and perceived BCS as benefit (Azaiza et al., 2010). Conversely, another study conducted among Arabic women showed that even with sufficient knowledge of BCS, their participation in screening activities remains low (Donnelly et al 2013a).

Although, BCS in Oman is offered freely at MOH hospitals and from the MMU, which is supported by the OCA, BCS is still not a national policy for screening in women of a certain age or for those who are at more risk of BC (Oman Cancer Association, 2019). Knowledge of guidelines is considered to be key for consistency, and women who are knowledgeable of examination recommendations for their age group or risk factors are better able to control and actively undergo BCS (Anderson et al., 2008). Currently, BCS is part of national healthcare policy in the UK, and women aged 50 to 69 are invited for a 2-view digital mammography every 3 years (Pashayan et al., 2018). 

Lack of implementation of a national policy for BCS in Oman might contribute to the low number of women reportedly attending BCS. Less than half of women in the current study knew where to go for BCS, including women with a family history of BC who should undergo early BCS. Lack of a centrally organized invitation or follow-up system is a common problem in most Arabic countries, and BCS remains as opportunistic for women who are self-motivated or referred by their doctors (El Saghiret al., 2007). Notably, many women in this study stated that they were not advised by doctors to attend BCS. Lack of knowledge among healthcare providers has been found to limit the enthusiasm of the patients to engage in screening activities; women are more likely to attend BCS when their healthcare providers encourage them (Borrayo et al., 2009). Doctors in the hospitals and LHCs in Oman are currently involved in providing clinical service, with less time for healthcare education for their patients, such as advising patients to attend BCS. 

Most women in this study were positive toward BCS, as they disagreed with negative statements, such as lower levels of reliability or less effectiveness, painfulness or belief in fate which prevented them from attending BCS. Globally, most participants in previous studies held positive attitudes toward cancer screening (Kamposioras et al., 2007; Schwartz et al., 2004). Visiting a doctor for health problems within the past six months, having a personal or family history of cancer were associated with more favourable attitudes and motivation to take part in preventive activities, such as screening (Straus et al., 2005; Cullati et al., 2009). Furthermore, positive attitudes and behaviours are consistent with most health psychology models and these could be used to promote BCS in Oman, as women who are more familiar with screening have less fear or wariness of screening procedures (Cullati et al., 2009). However, cancer fatalism or the belief that death is inevitable when cancer is present has a strong correlation to patient perceived benefit of screening, and whether one has the ability to affect cancer outcomes (Champion et al., 2004). 

An interesting finding from the current study was that women who underwent BCS in the past were still fate to have BC as undergoing screening does not always prevent the occurrence of BC. Previous uncomfortable experiences with BCS and incorrect beliefs, such as mammography is hazardous to health and painful, were important predictive barriers to BCS among Arabic women (Al-Zalabani et al., 2018; Azaiza and Cohen, 2006). Many of the women in the current study who had previously undergone BCS stated that they had experienced more pain and thought that screening was not as effective as compared to women who had never had BCS, which may support that there might be some element of discomfort with BCS in Oman. 

The women in the current study stated that they were getting high levels of BCS from social media. Formal education in Oman was only really initiated 50 years ago and the use of social media is expanding rapidly. Although there is currently a debate surrounding the effectiveness of social medial to promote disease prevention, the advantage of using social media is based on its capacity to reach a large audience or targeting specific audiences almost instantaneously using cost-effective methods (Neiger et al., 2012). Most of the women in this study indicated that traditional methods of providing medical education, such brochures and posters which are available at hospitals and LHCs, are not as effective to promote BCS. The majority of the Omani population are of a younger age and are educated; social media has become popular and an accessible source, with greater chances of causing an impact on screening behaviours and promoting health-related behaviours, such as BCS (Hornik et al., 2013). 

The most reported personal-related barriers for BCS for the women in this study were being embarrassed of BE, fear of a BC diagnosis and worries of the side effects from cancer treatments (mastectomy, chemotherapy, radiotherapy). Fear of cancer diagnosis or treatment side effects could act as barriers for BCS. A study conducted among Muslim women in Lebanon showed that one of the barriers to BCS was the belief that there was no cure for BC if diagnosed (Azaiza and Cohen, 2006). A study conducted among Omani women who were diagnosed with BC showed that women experienced several psychosocial stressors, such as worry of death, isolation, side effects of treatment, cancer spreading and the fear that cancer could interfere with their daily life and family responsibilities (Al-Azri et al., 2014). Conversely, other studies have shown that worries of a BC diagnosis could motivate BCS behaviours, when women perceive benefits of screening (Azami-Aghdash et al., 2015; Hay et al., 2006).

The findings from this study were that women are embarrassed of physical examination when attending a BCS, particularly with a male doctor; this is related to Omani. As with most Arabic countries, Oman is a conservative Muslim society and many women feel embarrassed if the breast examination is to be conducted by a male doctor. Studies in other Arabic countries support this, less religious or Christian women who underwent mammography screening and CBE were less likely than Muslim women to feel discomfort and embarrassed (Azaiza et al., 2010; Azaiza and Cohen, 2006). However, the assumption of women that there will be female doctors or staff available during their visits to conduct BCS is unfounded, as most of staff working in this field are female and the government always takes into consideration the cultural aspects when providing such services. 

In conclusion, although the majority of women in Oman were aware that BCS is available and needed, less than half of them knew where the screening was conducted. The work done by the OCA to promote awareness of BCS is very important, particularly with regards to the MMU which travels around the country. Stressing that BCS as an important necessity rather than a choice and, emphasizing the significance of adherence to yearly mammograms using social media could help to improve BCS awareness. Thus, more efforts are still needed to educate women in the community that BCS is also available at MOH hospitals and free of charge. A national policy or guideline for BCS in Oman could help healthcare professionals to advise women to attend. Furthermore, considering calling women of a certain age, sending reminders, particularly to women who are more at risk of BC, could also improve the BCS attendance. 

The most common personal-related barriers to BCS were fear of BC diagnosis and cancer treatments. More future efforts are needed to tackle women’s poor perception of BCS and rectify the current socially prevalent misconceptions about BC and screening. The emphasis should be focused on abating BC fears and stress to women that early BCS and detection leads to higher survival and remissions rates. Integrating educational campaigns and targeting female school and university students could help to raise awareness among the younger generation of the benefit of BCS and decrease the social stigma and misconceptions of BC and BCS. Notably, there is currently a strong emotional response regarding the issues of threats, benefits, fatalism and fear which are dependent on several concepts which need further investigations. Finally, availability of female staff who can speak Arabic and communicate well is important to reassure women and decrease their fears of attending BCS. 

*Limitations*

This study has some limitations. Firstly, the study was conducted among a small sample of Omani women attending the SQUH teaching hospital in Muscat, which could affect the generalisability of the results. Secondly, as the sample was hospital-based, there can be limited extrapolation of the findings to other women in Oman due to the possibility of response bias. Thirdly, 50% of the women involved in the study were between 25–40 years old, an age group which does not represent the target population for BCS. Fourthly, although the questionnaire underwent a test for reliability which demonstrated a satisfactory Cronbach’s alpha (α) coefficient, we cannot guarantee the results of other tests, such as validity, which were beyond the scope of this research. Finally, although the medical students who were involved in data collection for this study were trained to be neutral when helping illiterate patients, there could have been some disparities between their spoken statements compared to the written items of the questionnaire, potentially resulting in differences in responses between literate and illiterate respondents.
